# Supplementary material for: Environmental conditions shape the nature of a minimal bacterial genome
Source: Nat Commun. 2019 Jul 15;10:3100. doi: 10.1038/s41467-019-10837-2 (PMC6629657; doi:10.1038/s41467-019-10837-2)
Supplement: Supplementary file 1 — Supplementary Information [file 41467_2019_10837_MOESM1_ESM.pdf]

Environmental conditions shape the nature of a minimal bacterial genome

Magdalena Antczak, Martin Michaelis*, Mark N Wass*
School of Biosciences, University of Kent, Canterbury, Kent, CT2 7NJ, UK
*to whom correspondence should be addressed: m.n.wass@kent.ac.uk
m.michaelis@kent.ac.uk

Supplementary Material

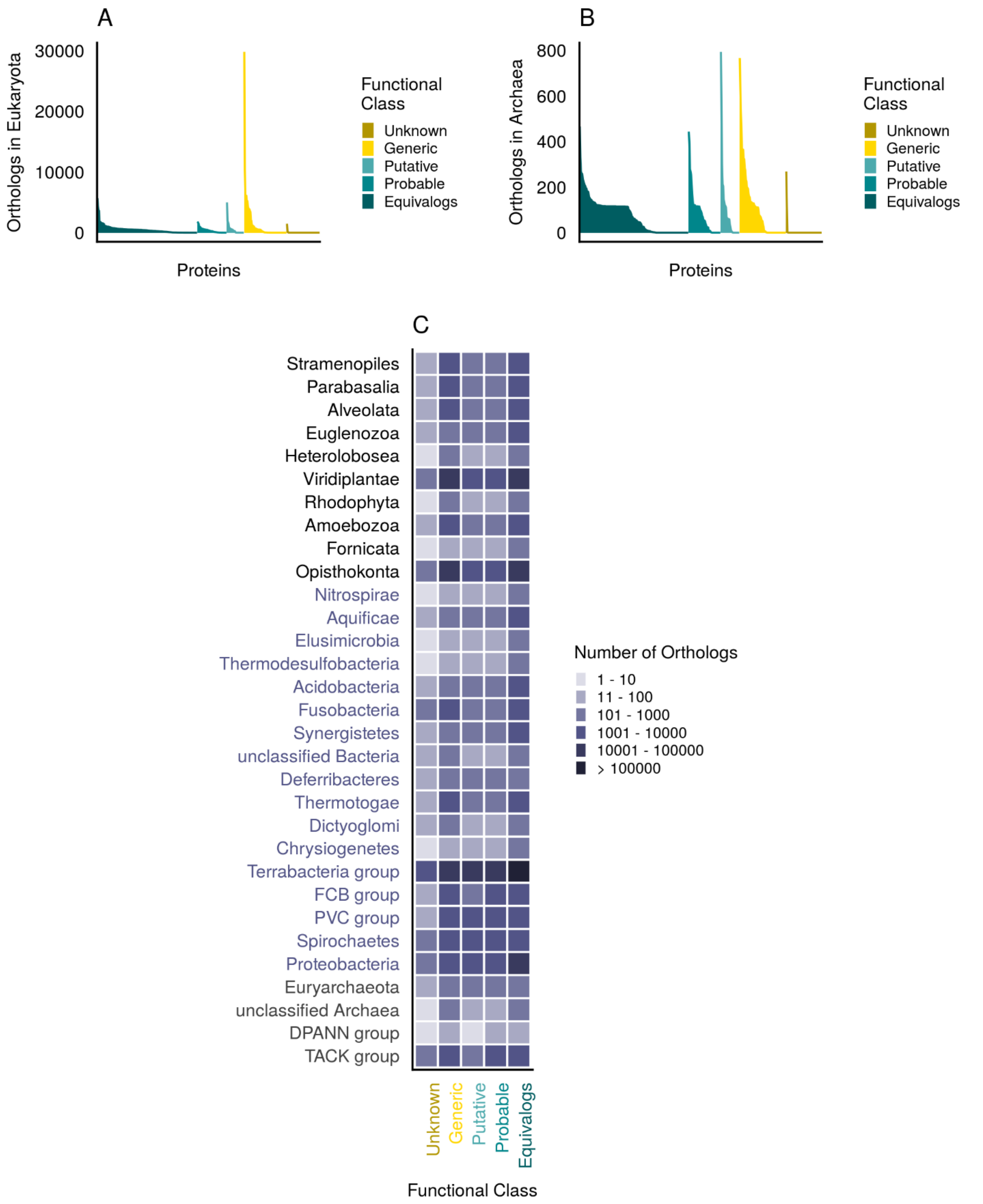

Supplementary Figure 1. Orthologs of proteins in the minimal bacterial genome. The number of orthologs for each protein identified in A) archaea and B) eukaryota. Results for each functional class are represented by a different colour: golden for the Unknown functional class, yellow – Generic, light turquoise – Putative, turquoise – Probable, dark turquoise – Equivalog. C) Summary of the total number of orthologs identified across different phyla for each of the functional confidence groups. The names of phyla from eukaryota are displayed in black, bacteria in red and archaea in grey.

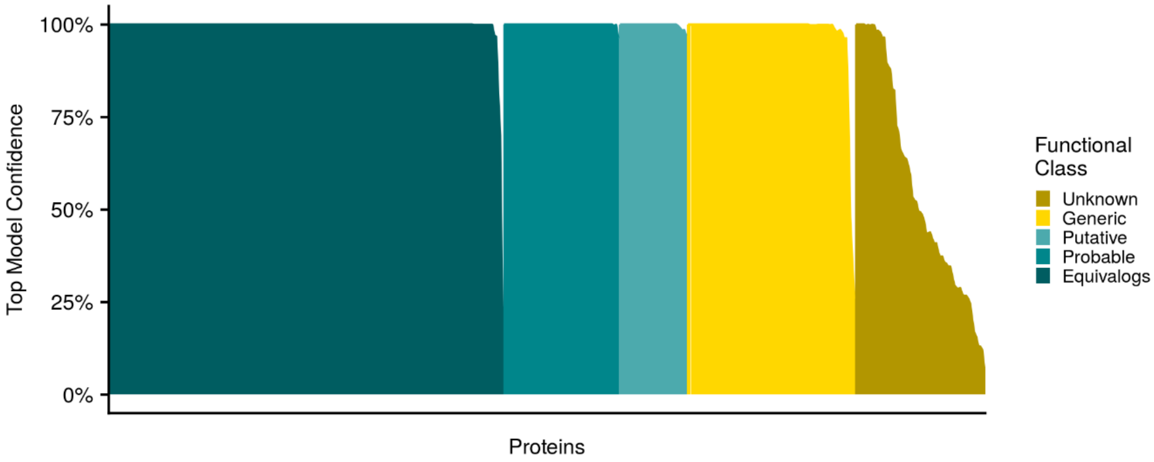

Supplementary Figure 2. Confidence of the top structural template identified by Phyre2. The confidence score (0-100) is shown for the top scoring template identified for each of the proteins in the minimal genome. The score indicates the confidence that the template protein sequence and the minimal genome protein sequence are homologs. Results for each functional class are represented by a different colour: golden for the Unknown functional class, yellow – Generic, light turquoise – Putative, turquoise – Probable, dark turquoise – Equivalog.

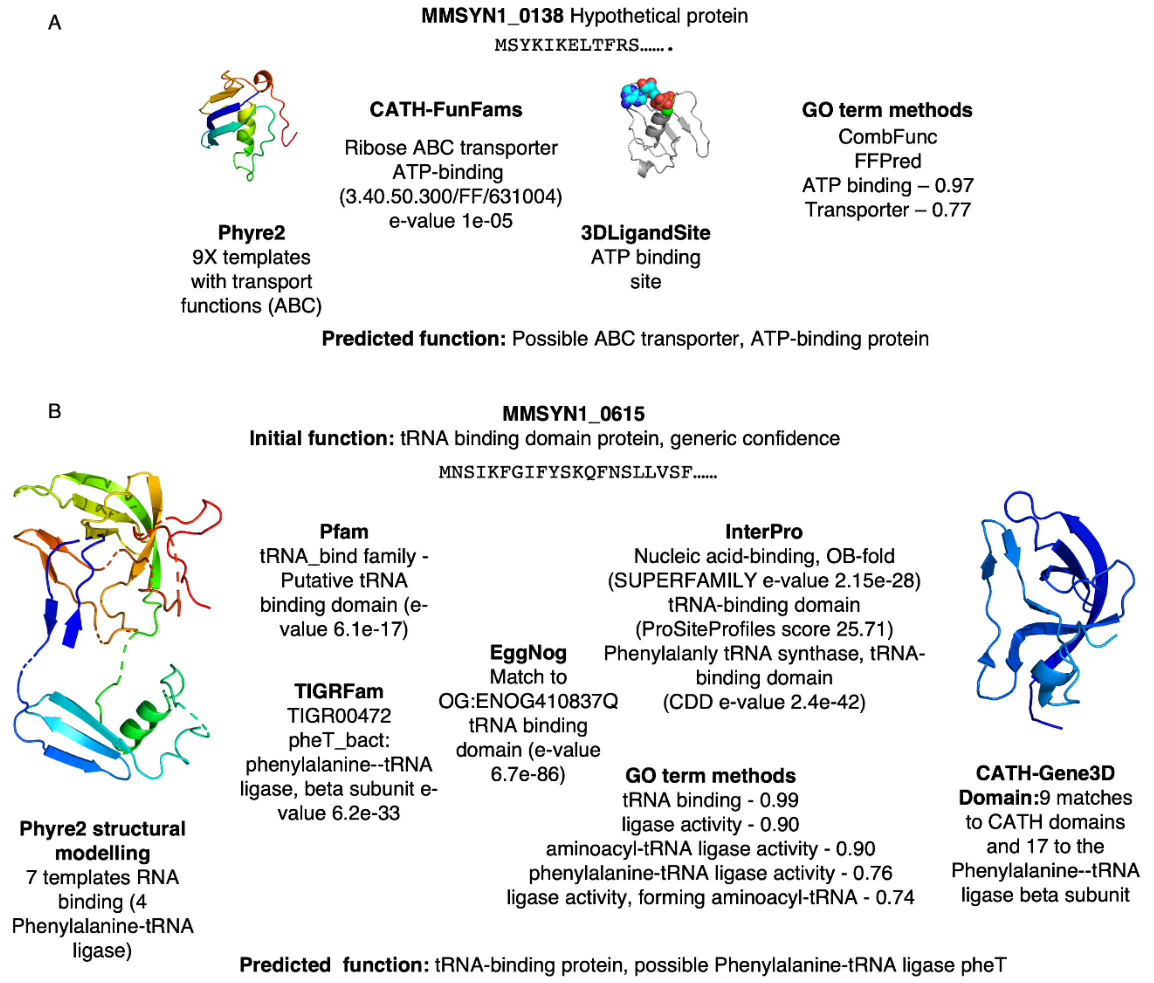

Supplementary Figure 3. Examples of proteins in the minimal bacterial genome that where it was difficult to predict their function. A) Protein MMSYN_0138 was previously completely uncharacterised and listed as a hypothetical protein. Predictions for MMSYN_0138 by multiple methods identify a relationship to ATP binding domains of ABC transporters but the functional residues involved in ATP binding are not conserved making this function less likely. B) Protein MMSYN_0615 was previously classified as a tRNA binding protein in the Generic confidence class. Multiple predictions suggest that it could be a Phenylalanine-tRNA ligase β subunit, however the β subunit in other bacteria typically contains around 800 residues, whereas MMSYN_0615 is only 202 residues. It therefore seems that tRNA binding is likely but the role of this function is not known.

A

MMSYN1_0165
Initial function: AmiC?
Confidence class: Generic

MKSTLKTKQEVLNLNSELLL......

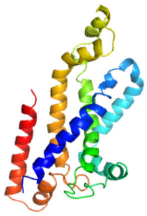

TIGRFam
nickel_nikB: nickel ABC transporter, permease subu (e-value 4e-22)

Pfam
Binding-protein-dependent transport system inner membrane component (e-value 1.1e-22)

TrSSP
Amino acid transporter

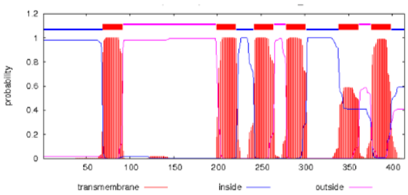

TMHMM
Predicted 6 TM helices

GO term methods
Multiple high confidence predictions associated with transmembrane transporter functions

EggNog
Match to OG: ENOG41082QK
ABC transporter (Permease (e-value 1.85e-104)
Predicted gene: oppB

Phyre2 structural modelling
Several confident matches to templates of ABC transporter, permease

CATH-FunFams
Oligopeptide ABC transporter permease (1.10.3720.10/FF/58662) e-value 1.7e-21

InterPro
ABC transporter type 1, transmembrane domain MetI-like (CDD e-value 1.87e-12; ProSiteProfiles score 10.45)

Predicted function: Oligopeptide ABC transporter, permease OppB (AmiC)

B

MMSYN1_0166
Initial function: AmiD?
Confidence class: Generic

MKTKQLEQPDFSALLDSERE......

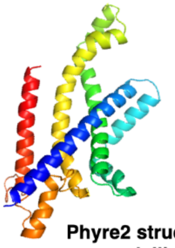

TIGRFam
nickel_nikC: nickel ABC transporter, permease subu (e-value 9.2e-25)

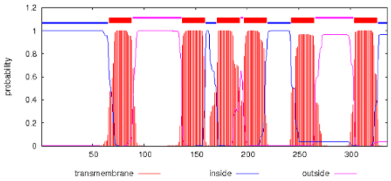

GO term methods
Multiple high confidence predictions associated with transmembrane transporter functions

EggNog
Match to OG:ENOG4107SI6
transport system permease protein (e-value 1.67e-146)
Predicted gene: oppC2

Phyre2 structural modelling
Several confident matches to templates of ABC transporter, permease

Pfam
Binding-protein-dependent transport system inner membrane component (e-value 6.4e-16)

TrSSP
Anion transporter

TMHMM
Predicted 6 TM helices

CATH-FunFams
Oligopeptide ABC transporter permease OppC (1.10.3720.10/FF/58605) e-value 1.2e-29

InterPro
ABC transporter type 1, transmembrane domain MetI-like (CDD e-value 7.43e-09; ProSiteProfiles score 17.83)

Predicted function: Oligopeptide ABC transporter, permease OppC (AmiD)

C

MMSYN1_0167
Initial function: AmiE?
Confidence class: Generic

MKNVILSIKDLVVKFRVRSK......

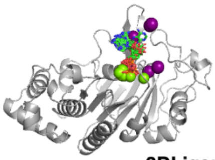

3DLigandSite
ATP binding site

EggNog
Match to OG:ENOG411CA8C
oligopeptide abc transporter atp-binding protein (4.4e-175)
Predicted gene: oppD

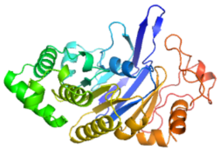

CATH-FunFams
Oligopeptide ABC transporter ATP-binding (3.40.50.300/FF/632531) e-value 3.5e-81

GO term methods
ATP binding - 0.99
ATPase activity - 0.92
peptide transport - 1.0

TIGRFam
Matches to ABC transporters, ATP-binding component

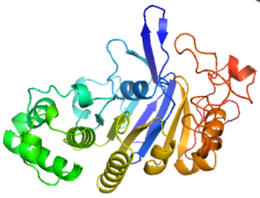

Phyre2 structural modelling
38 templates with ABC transporters, ATP-binding

Pfam
ATP-binding domain of ABC transporters (e-value 7.4e-09)
Oligopeptide/dipeptide transporter, C-terminal region (e-value 1.4e-10)

InterPro
ABC transporter-like (ProSiteProfiles score 19.48)
AAA+ ATPase domain (SMART e-value 3.2e-15)
ABC transporter, conserved site (ProSitePatterns)

Predicted function: Oligopeptide ABC transporter, ATP-binding protein OppD (AmiE)

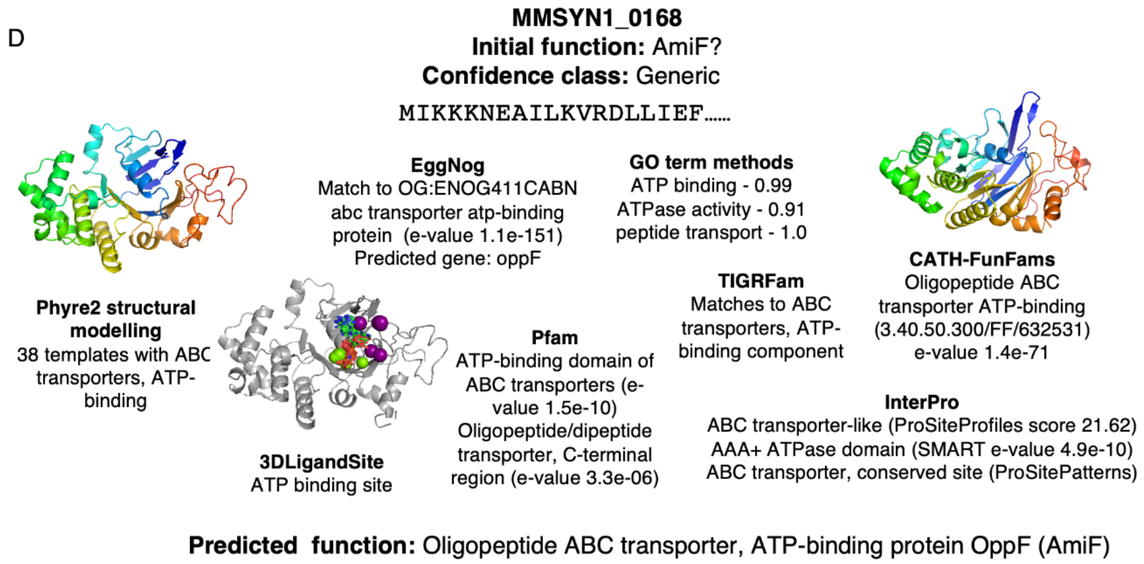

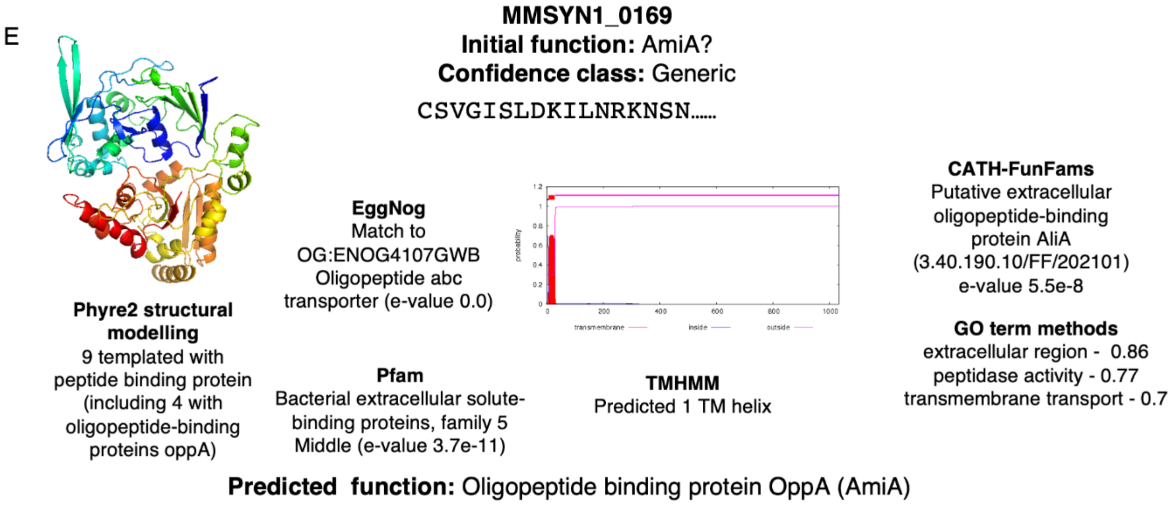

Supplementary Figure 4. Transporter function prediction for the OppABCDF operon. Multiple sources made confident prediction for the proteins of the oligopeptide transporter system OppABCDF (AmiABCDE). These proteins form an operon in the original M. mycoides subsp. capri and in the minimal genome. A) Permease OppB (AmiC) B) Permease OppC (AmiD) C) ATP-binding protein OppD (AmiE) D) ATP-binding protein OppF (AmiF) E) Oligopeptide binding protein OppA (AmiA).

A

MMSYN1_0195
Initial function: potCD or potHI?
Confidence class: Generic

MKKLLKRSYFAFVLLFIYAPIL......

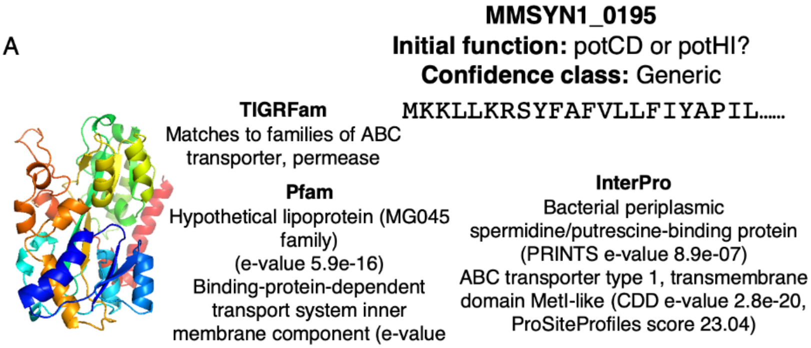

TIGRFam
Matches to families of ABC transporter, permease

Pfam
Hypothetical lipoprotein (MG045 family) (e-value 5.9e-16)
Binding-protein-dependent transport system inner membrane component (e-value 1.4e-10)

InterPro
Bacterial periplasmic spermidine/putrescine-binding protein (PRINTS e-value 8.9e-07)
ABC transporter type 1, transmembrane domain MetI-like (CDD e-value 2.8e-20, ProSiteProfiles score 23.04)

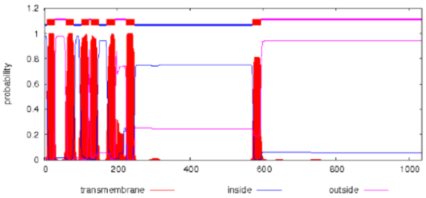

TMHMM
Predicted 7 TM helices

Phyre2 structural modelling
29 templates of ABC transporter (3 spermidine/putrescine ABC transporter potD)

EggNog
Match to OG: ENOG4105D38 putrescine abc transporter (e-value 1.1e-250)
Predicted gene: potC

GO term methods
Multiple high confidence predictions associated with transporter functions

CATH-FunFams
23 matches to functional families of ABC transporter permease
Spermidine/putrescine ABC transporter permease (1.10.3720.10/FF/58664) e-value 5.4e-50
Polyamine transport protein PotC (1.10.3720.10/FF/33149) e-value 3.5e-34

Predicted function: Spermidine/putrescine ABC transporter, permease and binding domains potCD

B

MMSYN1_0196
Initial function: potB or potG?
Confidence class: Generic

METKNLKDNNVIENKIINQDE......

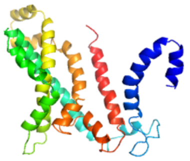

Phyre2 structural modelling
5 templates with permease functions (3 ABC transporters)

EggNog
Match to OG:ENOG41084AR spermidine putrescine ABC transporter (Permease (e-value 1.4e-95)
Predicted gene: potB

TMHMM
Predicted 6 TM helices

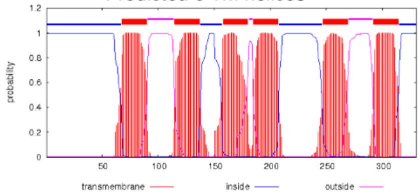

CATH-FunFams
24 matches to functional families of ABC transporter permease
Spermidine/putrescine ABC transporter permease (1.10.3720.10/FF/58725) e-value 5.1e-39
Polyamine transport protein PotB (1.10.3720.10/FF/4057) e-value 8.2e-26

TIGRFam
Matches to families of ABC transporter, permease

Pfam
Binding-protein-dependent transport system inner membrane component (e-value 1.5e-18)

InterPro
ABC transporter type 1, transmembrane domain MetI-like (CDD e-value 1.35e-14, ProSiteProfiles score 22.45)

GO term methods
Multiple high confidence predictions associated with transporter functions

Predicted function: Spermidine/putrescine ABC transporter, permease subunit potB

C

MMSYN1_0197
Initial function: potA or potF?
Confidence class: Generic

MFSWDLYIINPLLIVIWLIVA......

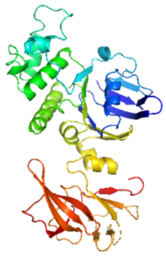

Pfam
ATP-binding domain of ABC transporters (e-value 1.5e-34)
Transport-associated OB (e-value 6.8e-07)

TIGRFam
TIGR01187 potA: polyamine ABC transporter, ATP-binding prote, e-value 2.5e-106

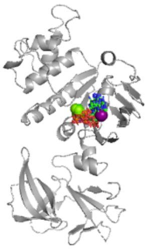

EggNog
Match to OG:ENOG410NDIN
Part of the ABC transporter complex PotABCD involved in spermidine putrescine import (e-value 8.1 e-163)
Predicted gene: potA

GO term methods
ATP binding - 0.99
polyamine-transporting ATPase activity - 0.99
ATP-binding cassette (ABC) transporter complex - 0.94
putrescine/spermidine transmembrane transport - 0.75

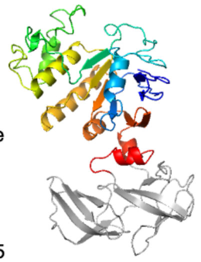

Phyre2 structural modelling
36 templates with ABC transporters, ATP-binding

3DLigandSite
ATP binding site

InterPro
ABC transporter, spermidine/putrescine import ATP-binding protein, PotA (ProSiteProfiles score 139.2)
AAA+ ATPase domain (SMART e-value 1.4e-18)
ABC transporter, conserved site (ProSitePatterns)

CATH-FunFams
30 matches to functional families of ABC transporter, ATP-binding component

Predicted function: Spermidine/putrescine ABC transporter, ATP-binding subunit potA

Supplementary Figure 5. Transporter function prediction for the potABCD operon. Multiple sources made confident prediction for the of the spermidine/putrescine transporter system

potABCD were moved to the Putative class based on function predicted using confident results from multiple sources. These proteins form an operon in the original M. mycoides subsp. capri and in the minimal genome. A) Permease subunit potCD B) Permease subunit potB C) ATP-binding subunit potA.

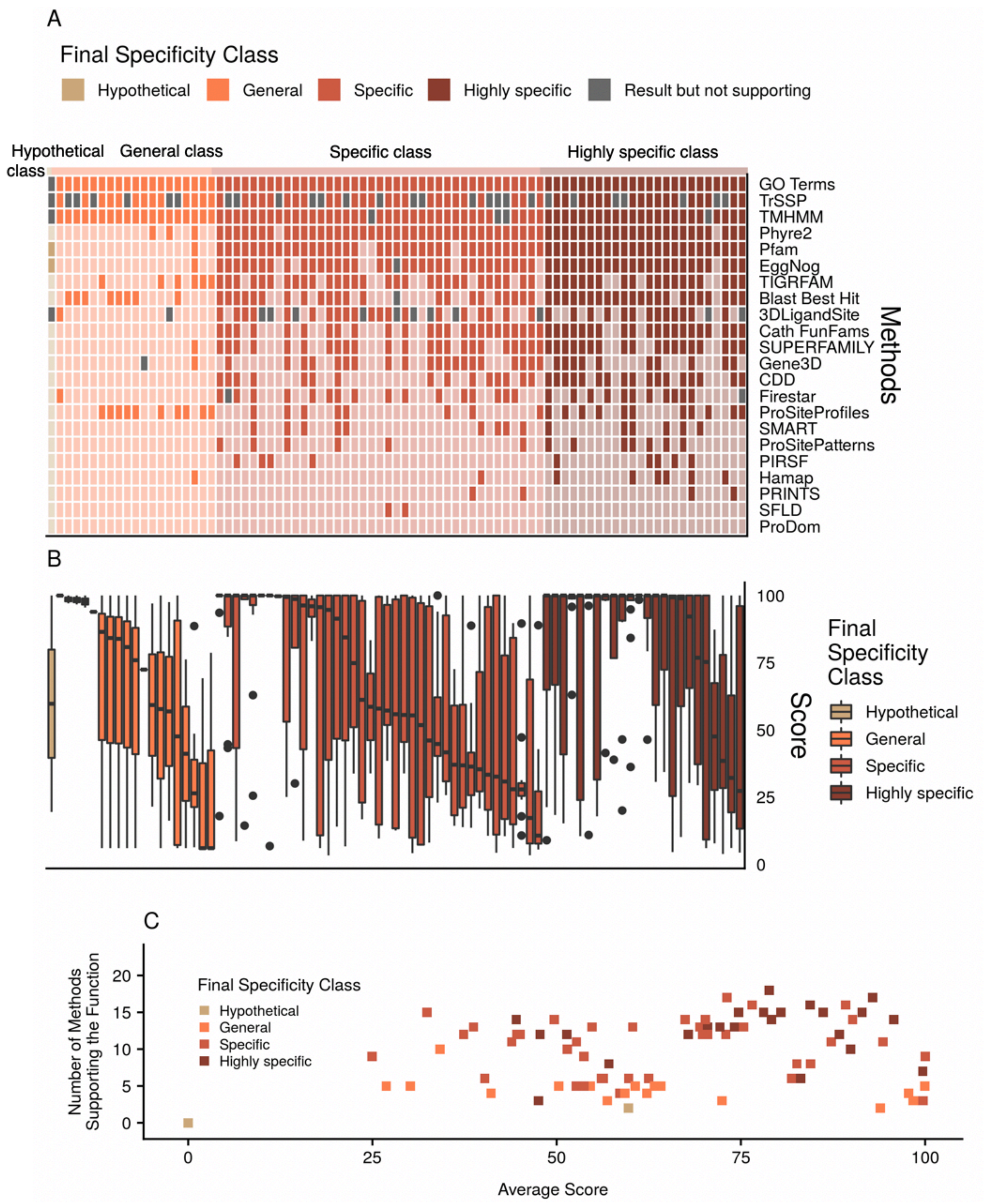

Supplementary Figure 6. Functional annotations where confidence was increased. This figure shows the proteins of unknown function that remained in the same specificity class. Results for each specificity class are represented by a different colour: beige for the Hypothetical specificity class, orange – General, light brown – Specific and dark brown – Highly specific. A) Each column represents a protein in the minimal genome and the squares

show the methods that made predictions (darker colours indicate support of the final prediction), grey squares indicate predictions that did not support the function, light squares indicate that a method did not make a prediction. Proteins are grouped by their initial specificity class (Hypothetical, General, Specific and Highly specific) B) Boxplot showing the distribution of scores associated with the annotated functions. Proteins are grouped by their initial specificity class. Horizontal lines represent the median, the lower and upper hinge show respectively first quartile and third quartile, and lower and upper whisker include scores from first quartile to (distance between the first and third quartile)*1.5 (for lower whisker) and from third quartile to (distance between the first and third quartile)*1.5 (for upper whisker). Any scores outside of these intervals are shown as points (outliers). C) Number of methods supporting the function and the average score. Each point represents a protein. Note that the point at 0,0 represents multiple proteins classed as Hypothetical where it was not possible to assign any function.

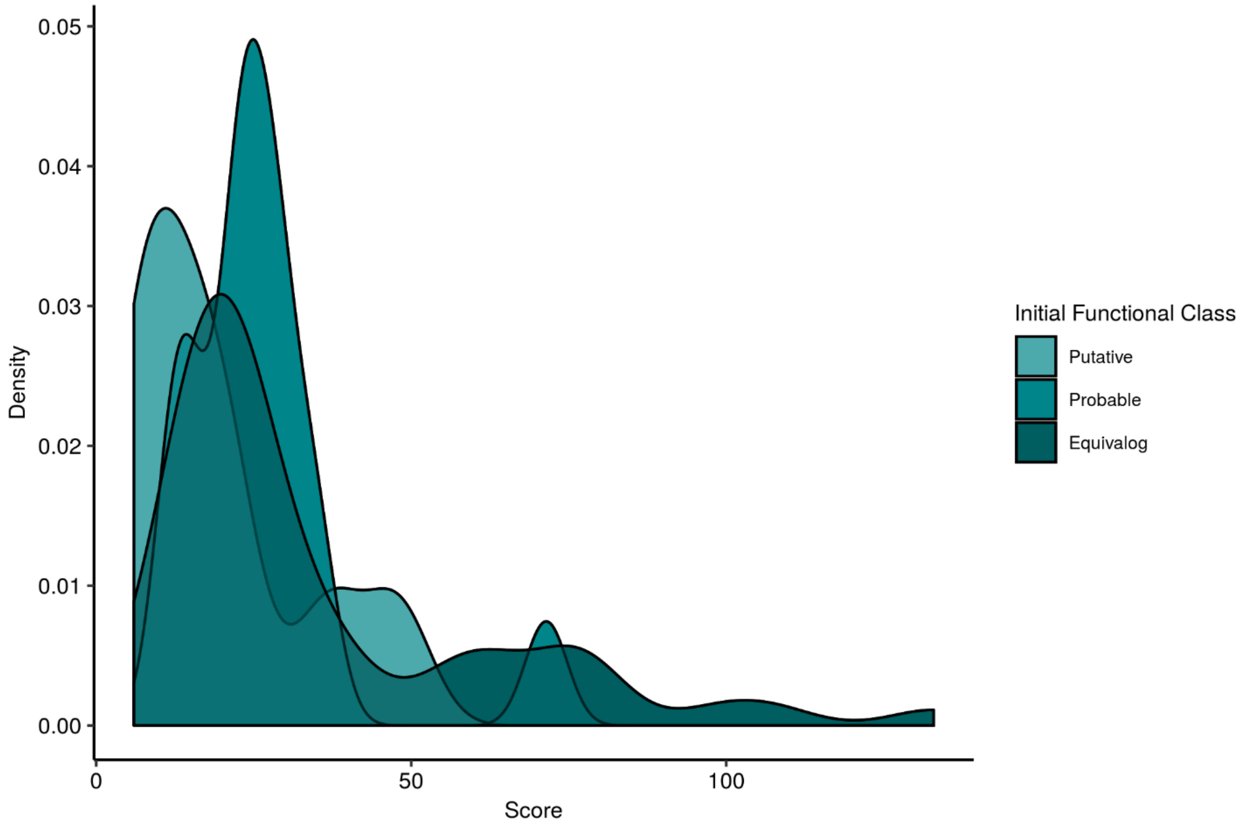

Supplementary Figure 7. Distribution of scores for matches to HAMAP. This the scores for HAMAP results for the minimal genome proteins of known function (Putative, Probable and Equivalog functional classes) are plotted. Results for each functional class are represented by a different colour: light turquoise – Putative, turquoise – Probable, dark turquoise – Equivalog.

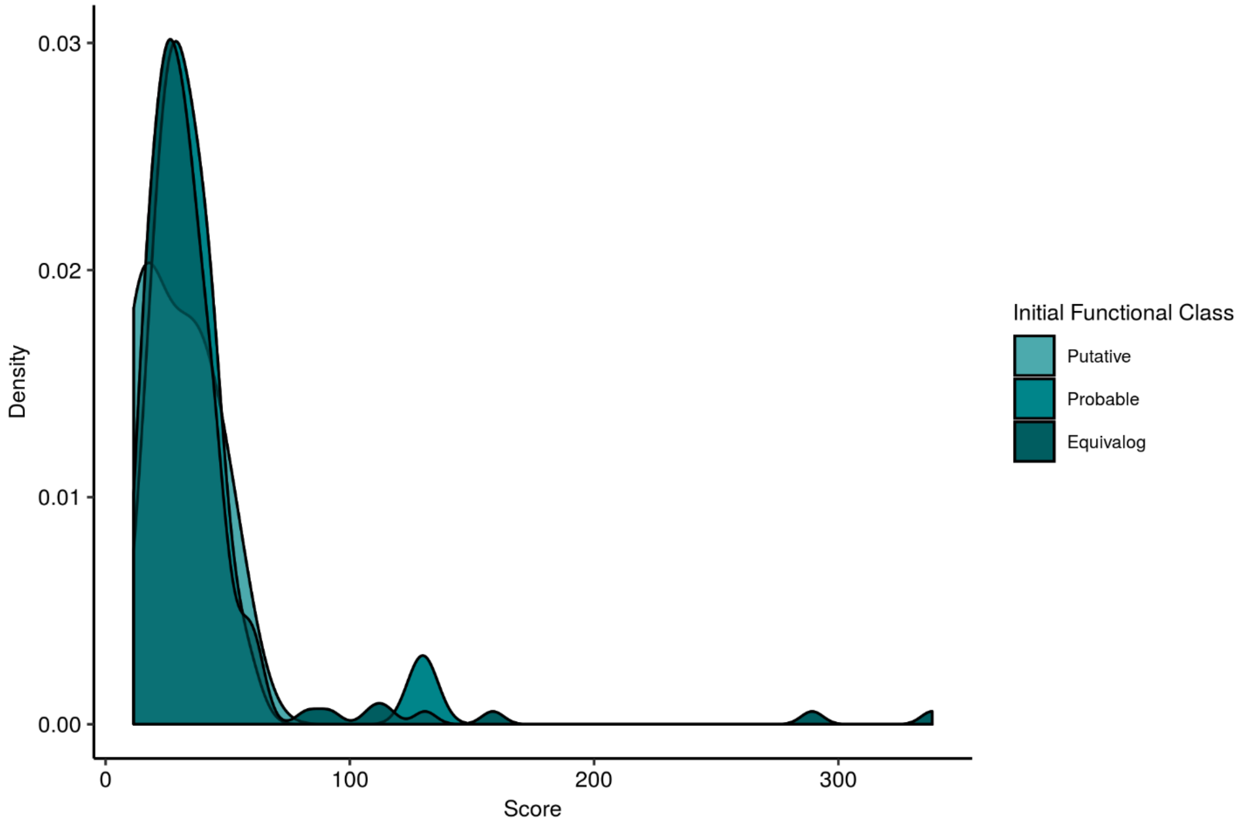

Supplementary Figure 8. Distribution of scores from ProSiteProfiles results. This figure plots the scores for ProSiteProfiles results for the minimal genome proteins of known function (Putative, Probable and Equivalog functional classes). Results for each functional class are represented by a different colour: light turquoise – Putative, turquoise – Probable, dark turquoise – Equivalog.

Supplementary Tables

General	Specific	Highly specific
Transcription factor	Transcriptional regulator, RpiR family	whiA; Sporulation transcription regulator WhiA
Ribosomal protein	Ribosomal protein L7Ae/L30e family	rpmH; 50S ribosomal protein L34
Transmembrane protein, likely a transporter	ABC transporter, ATP-binding protein	oppD; Oligopeptide ABC transporter, ATP-binding protein
Membrane metallopeptidase	Transmembrane peptidase, C39 family	pepQ; Xaa-Pro dipeptidase
DNA-binding protein	ATP-dependent DNA helicase	polA; DNA polymerase I

Supplementary Table 1. Examples of protein functions for the specificity classes.

Methods	Number of proteins				Percentage			
	Yes (final)	Yes (general)	No	No prediction	Yes (final)	Yes (general)	No	No prediction
eggNOG-Mapper	55	22	1	71	37%	15%	1%	48%
GO Terms	53	80	0	16	36%	54%	0%	11%
Phyre2	53	32	0	64	36%	21%	0%	43%
BLAST against UniProt top match	51	20	1	77	34%	13%	1%	52%
Pfam	49	34	0	66	33%	23%	0%	44%
CATH FunFams	45	16	2	86	30%	11%	1%	58%
TIGRFAM	41	24	1	83	28%	16%	1%	56%
InterPro ProSiteProfiles	21	12	0	116	14%	8%	0%	78%
InterPro CDD	21	21	0	107	14%	14%	0%	72%
InterPro SUPERFAMILY	21	40	0	88	14%	27%	0%	59%
TrSSP	14	71	48	16	9%	48%	32%	11%
InterPro Gene3D	14	28	1	106	9%	19%	1%	71%
InterPro PIRSF	7	4	0	138	5%	3%	0%	93%
InterPro HAMAP	7	1	0	141	5%	1%	0%	95%
InterPro SMART	7	11	0	131	5%	7%	0%	88%
TMHMM	6	122	5	16	4%	82%	3%	11%
InterPro ProSitePatterns	4	12	0	133	3%	8%	0%	89%
InterPro PRINTS	3	4	0	142	2%	3%	0%	95%
InterPro SFLD	2	1	0	146	1%	1%	0%	98%
3DLigandSite	0	44	20	85	0%	30%	13%	57%
Firestar	0	35	2	112	0%	23%	1%	75%
InterPro ProDom	0	1	0	148	0%	1%	0%	99%

Supplementary Table 2. Comparison of the predictions made by individual methods and the final annotation assigned by the combination of methods. For each individual method we counted the predictions that agreed with the final annotation assigned to the protein (column yes – final) and if they more generally agreed with the assigned function (yes – general).

Method 1	Method 2	Number of common proteins	Percentage
EggNOG	BLAST - UniProt	38	25.5
EggNOG	Pfam	31	20.81
EggNOG	Phyre2	30	20.13
Phyre2	Pfam	28	18.79
Phyre2	BLAST - UniProt	26	17.45
BLAST - UniProt	Pfam	24	16.11
EggNOG	GO Terms	14	9.4
GO Terms	Phyre2	13	8.72
GO Terms	BLAST - UniProt	12	8.05
GO Terms	Pfam	9	6.04

Supplementary Table 3. Common predictions made by the five methods with greatest agreement with the final annotation. For each pair of methods the number of proteins where both methods make the same prediction as the final annotation is shown.
