# Supplementary material for: Environmental conditions shape the nature of a minimal bacterial genome
Source: Nat Commun. 2019 Jul 15;10:3100. doi: 10.1038/s41467-019-10837-2 (PMC6629657; doi:10.1038/s41467-019-10837-2)
Supplement: Supplementary file 11 — Description of Additional Supplementary Files [file 41467_2019_10837_MOESM11_ESM.docx]

**Title:** Supplementary Data File 1.
**Description:** Orthologs of the minimal genome proteins identified using eggNOG-Mapper.

**Title:** Supplementary Data File 2.
**Description:** Domains identified in the minimal genome proteins. Results are shown for search against the Pfam and TIGRFAM databases of protein families.

**Title:** Supplementary Data File 3.
**Description:**  Structural modelling of the minimal genome proteins. Results for modelling of the proteins using the Phyre2 server.

**Title:** Supplementary Data File 4.
**Description:** Membrane protein predictions for the minimal genome proteins.

**Title:** Supplementary Data File 5.
**Description:** Inferred functions of the proteins encoded by the minimal bacterial genome. The original annotation and the predicted functions from the analysis performed here are shown.

**Title:** Supplementary Data File 6.
**Description:** Gene Ontology based function predictions for the proteins encoded by the minimal genome.

**Title:** Supplementary Data File 7.
**Description:** Comparison of the predicted functions of the minimal genome proteins with predictions made by Danchin and Fang.

**Title:**  Supplementary Data File 8.
**Description:** Interpro predictions for the proteins encoded by the minimal bacterial genome.
